# Integrative systems analysis reveals heat-induced functional divergence in cultivated and wild tomato genotypes

**DOI:** 10.1186/s12870-026-09807-w

**Published:** 2026-08-28

**Authors:** Eray Şimsek, Kübra Yıldız, Sertan Çevik, M. Aydın Akbudak

**Affiliations:** 1 Department of Plant Protection, Faculty of Agriculture, Harran University, Şanlıurfa, Turkey; 2 Department of Molecular Biology and Genetics, Faculty of Science, Harran University, Şanlıurfa, Turkey; 3 Department of Agricultural Biotechnology, Akdeniz University, Antalya, Turkey

**Keywords:** *Solanum lycopersicum*, Growth–defense trade-off, Thermotolerance, Correlation network analysis, Multivariate integration, Antioxidant defense, Wild germplasm

## Abstract

Extreme heat events driven by global warming increasingly threaten the productivity of tomato (*Solanum lycopersicum* L.). While thermotolerance is often evaluated through individual physiological traits, resilience emerges from coordinated, multi-level regulation across growth dynamics and cellular stress responses. Here, we analyzed a diverse and contrasting panel of cultivated and wild tomato genotypes to resolve multidimensional stress-response strategies. Morphological, physiological, and biochemical traits were systematically quantified and integrated using the Stress Tolerance Index (STI), Membership Function Value (MFV), and correlation network analysis.

Heat exposure induced pronounced, genotype-dependent divergence in biomass retention, membrane stability, pigment integrity, osmotic adjustment, and antioxidant capacity. Sensitive accessions (e.g., *S. chilense* PI 251313) exhibited severe growth suppression, chlorophyll loss, and elevated lipid peroxidation. In contrast, the commercial cultivar *S. lycopersicum* cv. İksir maintained biomass and structural stability, whereas the wild accession *S. pimpinellifolium* PI 365957 displayed enhanced antioxidant coordination and membrane protection.

Network analysis revealed a heat-induced shift from growth-centered regulation to a tightly interconnected stress-response module, with strong associations among proline, superoxide dismutase, and catalase. Multivariate integration consistently ranked *S. lycopersicum* cv. İksir and *S. pimpinellifolium* PI 365957 as the most thermotolerant genotypes at the vegetative stage under acute thermal stress (45 °C), highlighting complementary adaptive strategies across cultivated and wild backgrounds.

Collectively, these findings establish thermotolerance as an emergent systems property and identify wild relatives as valuable reservoirs of coordinated stress-adaptive traits for climate-resilient tomato breeding.

**Supplementary Information:**

The online version contains supplementary material available at https://doi.org/10.1186/s12870-026-09807-w.

## Introduction

Global warming has intensified both the frequency and duration of extreme temperature events, establishing heat stress as one of the most critical abiotic constraints threatening global food security. Tomato (*Solanum lycopersicum* L.), a crop of major economic and nutritional significance, is particularly sensitive to elevated temperatures. In cultivated tomato, the optimal growth temperature is approximately 25/20°C (day/night), whereas sustained exposure above 35 °C markedly impairs physiological performance [1–3]. In the Mediterranean region—responsible for nearly 25% of global fresh tomato production—greenhouse temperatures during summer can escalate to 45–55 °C, subjecting plants to severe thermal stress. Prolonged exposure to such extreme conditions disrupts metabolic homeostasis, compromises membrane integrity, and ultimately reduces productivity and yield stability [4].

The photosynthetic apparatus represents one of the earliest and most thermosensitive targets of heat injury, as elevated temperatures disrupt chloroplast structure and compromise photochemical efficiency [5, 6]. Alongside these primary photochemical disruptions, acute heat stress severely alters cellular water relations and carbon assimilation dynamics, forcing the plant into a state of severe metabolic imbalance [7–9]. When this fundamental physiological equilibrium fails, it inevitably triggers the accelerated overproduction of reactive oxygen species (ROS), including superoxide radicals (O_2_^•−^), hydrogen peroxide (H_2_O_2_), and hydroxyl radicals (•OH) within the cellular compartments [10–12].

At the cellular level, elevated temperatures disturb membrane fluidity and accelerate the production of reactive oxygen species (ROS), leading to oxidative stress [10]. Excess ROS promotes lipid peroxidation, generating malondialdehyde (MDA), a widely accepted biomarker of membrane damage [11, 12]. Simultaneously, electrolyte leakage reflects the functional loss of membrane semi-permeability. Increasing evidence indicates that membrane stability constitutes a critical physiological boundary separating tolerant from susceptible tomato germplasm [13]. Thus, thermotolerance is not solely defined by photosynthetic preservation but also by the plant’s capacity to coordinate oxidative defense and maintain structural integrity under hyperthermic conditions.

To mitigate osmotic and oxidative disturbances, plants activate an integrated defense network involving compatible solutes and antioxidant enzymes. Proline accumulation is one of the most common heat-induced responses, functioning in osmotic adjustment, ROS detoxification, and stabilization of proteins and membranes [10]. Enzymatic antioxidants such as superoxide dismutase (SOD) and catalase (CAT) sequentially detoxify superoxide radicals and hydrogen peroxide, thereby preventing oxidative bursts from escalating into irreversible cellular damage. The extent to which osmolyte accumulation and antioxidant enzyme activities are synchronized appears to be a decisive determinant of survival under thermal stress. However, substantial genotype-specific variation exists in how these pathways are modulated, particularly between cultivated varieties and wild relatives.

Although vegetative resilience is essential, the reproductive stage remains the most heat-sensitive developmental phase in tomato. Elevated temperatures impair pollen viability, pollen tube growth, carbohydrate metabolism in anthers, and ultimately fruit set [14, 15].

Modern commercial cultivars often derive from a relatively narrow genetic base, limiting adaptive plasticity under rapidly changing climatic conditions [16, 17]. Wild tomato relatives such as *S. pimpinellifolium*, *S. galapagense*, *S. habrochaites*, and *S. arcanum* represent invaluable reservoirs of stress-adaptive alleles [18]. Screening programs under controlled and field heat regimes have identified tolerant accessions capable of maintaining photosynthetic efficiency, membrane stability, or reproductive performance [6, 19]. Advanced breeding tools—including backcross inbred lines (BILs), quantitative trait locus (QTL) mapping, and integrative phenotyping—facilitate the introgression of these complex, polygenic tolerance mechanisms into elite germplasm [20, 21].

Despite this progress, thermotolerance remains insufficiently understood as a systems-level phenomenon. Many studies evaluate individual physiological parameters in isolation, whereas heat resilience emerges from coordinated reprogramming across morphological, physiological, and biochemical layers. Therefore, integrative multivariate approaches are essential to elucidate genotype-specific response architectures and to identify germplasm combining growth stability with robust cellular defense.

In this study, we evaluated thirteen cultivated and wild *Solanum* genotypes under acute heat stress conditions (45 °C) to (i) quantify growth-related performance, (ii) assess photosynthetic pigment stability and water status, (iii) determine membrane integrity through lipid peroxidation analysis, (iv) characterize osmotic adjustment and antioxidant enzyme responses, and (v) integrate these traits using Stress Tolerance Index (STI), correlation network analysis, and Membership Function Value (MFV) ranking.

Our results revealed a clear genotype-dependent divergence in thermotolerance strategies, where certain cultivated and wild accessions maintained biomass through coordinated osmotic adjustment and antioxidant activation, while sensitive genotypes exhibited membrane destabilization and metabolic decoupling. Multivariate integration further demonstrated a heat-induced transition from growth-centered regulation to a defense-oriented physiological network.

## Materials and methods

### Plant material and growth conditions

The plant material evaluated in this study comprised eleven wild *Solanum* accessions obtained from the USDA Germplasm Resources Information Network (USDA-GRIN), and two commercial *S. lycopersicum* cultivars, ‘Kamenta’ (determinate) and ‘İksir’ (indeterminate) obtained from seed supplier “Hazera” and “Semillas Fitó Türkiye”, respectively from Antalya, Türkiye. All selected materials belong to the *Lycopersicon* group (the tomato clade) within the genus *Solanum*. The commercial cultivars were included as reference standards based on their established agronomic performance and reported thermotolerance (Table 1). These 13 genotypes were strategically assembled to represent a broad genetic and phenotypic spectrum, based on the extensive diversity available in the USDA-GRIN collection. The panel encompasses both deeply divergent wild relatives (e.g., *S. pennellii*, *S. arcanum*, *S. pimpinellifolium*) and modern commercial cultivars (*S. lycopersicum*). For each available species, at least two genotypes were included, with a preference for accessions originating from different countries to further enhance genetic diversity. In addition, two commercial cultivars with documented heat tolerance were incorporated.

**Table 1:** Germplasm composition, taxonomic classification, and geographic origin of the tomato genotypes

Germplasm Type	Species	Accession / Cultivar	Geographic Origin
Reference Cultivar	*S. lycopersicum*	Kamenta (det.)	Commercial cultivar (Türkiye)
Reference Cultivar	*S. lycopersicum*	İksir (indet.)	Commercial cultivar (Türkiye)
Wild Accession	*S. arcanum*	PI 127828	Peru
Wild Accession	*S. arcanum*	PI 127829	Peru
Wild Accession	*S. galapagense*	PI 231257	Galápagos Islands (Ecuador)
Wild Accession	*S. galapagense*	PI 379042	Galápagos Islands (Ecuador)
Wild Accession	*S. pennellii*	PI 246502	Peru
Wild Accession	*S. pimpinellifolium*	PI 365918	Ecuador
Wild Accession	*S. pimpinellifolium*	PI 365957	Peru
Wild Accession	*S. cheesmaniae*	PI 379037	Galápagos Islands (Ecuador)
Wild Accession	*S. habrochaites*	PI 390513	Ecuador
Wild Accession	*S. habrochaites*	PI 390659	Peru
Wild Accession	*S. chilense*	PI 251313	Chile

This diverse panel was deliberately designed to capture contrasting evolutionary adaptations to thermal stress—ranging from the high baseline agronomic vigor of domesticated lines to the inherent extremophile resilience of wild accessions. Such an assembly maximizes the genetic variance required to comprehensively evaluate multidimensional thermotolerance mechanisms and facilitates the identification of robust genetic donors for future climate-resilient breeding programs.

For simplicity, these materials are hereafter collectively referred to as genotypes, reflecting their use as experimental units. The terms cultivar, accession, and wild relative will be used only when necessary to emphasize the specific origin or biological status of the plant material.

Seeds were sown in plug trays under controlled environmental conditions. After 21 days, uniform seedlings at the four-true-leaf stage were transplanted into 11 × 11 × 11 cm pots containing a peat: perlite mixture (2:1, v/v). Plants were acclimated for 10 days in a growth chamber at 27/25 ± 2 °C (day/night), 60% relative humidity, and a 12 h light/12 h dark photoperiod. Irrigation was applied every two days to maintain optimal substrate moisture.

### Experimental design and heat stress treatment

The experiment followed a completely randomized design (CRD) with three biological replicates per genotype. All experiments were conducted under strictly controlled growth chamber conditions to minimize background environmental variance. This stringent environmental control ensured that three independent biological replicates (*n* = 3) per genotype and treatment provided robust and sufficient statistical power to detect significant biochemical and physiological treatment effects. At the 5–6 true-leaf stage, plants were randomly assigned to control or heat stress treatments. Control plants were maintained at 27/25 ± 2 °C (day/night). Heat stress was imposed gradually to simulate field heatwave conditions. Temperature was increased by 2 °C per day for three days, followed by 3 °C per day until reaching 45/43°C (day/night). The acute temperature regime of 45 °C (day/night) was specifically selected to simulate the severe, episodic summer heatwaves anticipated under future global climate change scenarios. The peak temperature of 45 °C was deliberately selected to simulate the extreme, acute midday heatwave conditions characteristic of major tomato production and breeding regions, such as the Mediterranean Basin, where summer ambient temperatures frequently exceed 42 °C. Furthermore, under field conditions characterized by high solar radiation and restricted soil moisture, actual canopy microclimate temperatures can significantly exceed ambient air temperatures. Additionally, the application of a 45 °C threshold is consistent with several previous physiological studies designed to evaluate severe, acute heat stress tolerance in crop and model plants [22–25]. Therefore, applying this threshold provides a rigorous and realistic screening environment to identify true genetic resilience, ensuring that the selected germplasm can withstand the severe agricultural realities of future accelerated climate change scenarios. Relative humidity was maintained at 60% throughout the experiment. Plants were irrigated regularly to prevent drought effects. Stress exposure continued for two days after stabilization at the peak temperature.

### Growth Measurements and Relative Water Content (RWC)

Plant height was measured from the root collar to the shoot apex at harvest. Shoots were excised at soil level and fresh weight was recorded immediately (± 0.01 g).

For the determination of RWC, the youngest fully expanded leaves from the apex of each plant was excised, and its fresh weight (FW) was immediately recorded and RWC was determined following [26]. Fresh weight (FW) was recorded, leaves were hydrated overnight to obtain turgid weight (TW), and subsequently oven-dried at 70 °C for 24 h to determine dry weight (DW).

$$\mathrm{RWC}\left(\%\right)=\left[\left(\mathrm{TW}-\mathrm{DW}\right)/\left(\mathrm{FW}-\mathrm{DW}\right)\right]\times100$$

### Photosynthetic pigment analysis

Chlorophyll pigments were extracted using 80% (v/v) acetone, and the total chlorophyll, chlorophyll *a*, and chlorophyll *b* concentrations were determined spectrophotometrically according to the following equations formulated by [27]. Absorbance was measured at 645 and 663 nm.

The concentrations of chlorophyll *a* (Chl *a*), chlorophyll *b* (Chl *b*), and total chlorophyll were calculated using the standard equations formulated by Arnon (1949) [27]:

$$\mathrm{Chl}\:a=12.21{A}_{663}-2.81{A}_{645}$$

$$\mathrm{Chl}\:b=20.13{A}_{645}-5.03{A}_{663}$$

$$\mathrm{Total}\:Chl=17.3{A}_{645}+7.18{A}_{663}$$

Pigment concentrations were expressed as mg g⁻¹ fresh weight.

### Biochemical assays

To optimize protein and metabolite extraction from tomato leaf tissues, which are inherently rich in interfering phenolic compounds, samples were homogenized in ice-cold extraction buffers supplemented with 1% (w/v) polyvinylpyrrolidone (PVP). Lipid peroxidation was estimated by quantifying malondialdehyde (MDA) equivalents using the thiobarbituric acid (TBA) method [28]. The homogenate was reacted with TBA at 95 °C, and the absorbance of the resulting red chromophore was measured at 532 nm, subtracting the non-specific baseline absorption at 600 nm. Free proline content was determined via the acid-ninhydrin assay [29] where sulfosalicylic acid extracts were heated with ninhydrin and glacial acetic acid, extracted into toluene, and measured at 520 nm. For the enzymatic assays, Superoxide Dismutase (SOD) activity was quantified based on its ability to inhibit the photochemical reduction of nitroblue tetrazolium (NBT) at 560 nm [30] and Catalase (CAT) activity was assessed by monitoring the initial rate of hydrogen peroxide (H_2_O_2_) decomposition at 240 nm [31]. Total soluble protein was determined using the [32]. Enzyme activities were expressed per mg protein. To minimize artifactual errors and ensure high accuracy in enzymatic activity quantification -particularly avoiding the common pitfalls associated with non-specific NBT reduction as cautioned by Beyer and Fridovich (1987)- stringent quality control measures were implemented. All tissue homogenization and protein extractions were performed strictly at 4 °C to prevent enzyme denaturation. For the SOD assay, reaction mixtures were exposed to a standardized light intensity (300 µmol m⁻² s⁻¹) for precisely 15 min to guarantee uniform photochemical reduction, and all spectrophotometric readings (including CAT) were conducted immediately using freshly prepared buffer solutions across three independent biological replicates and technical triplicates.

### Statistical and integrative analyses

Morphological (growth) measurements were recorded from five independent biological replicates (n = 5) per genotype and treatment. All subsequent physiological and biochemical measurements were summarized from three independent biological replicates (n = 3) and are presented as mean ± standard error (SE) in the respective graphical representations. To evaluate the statistical significance of the observed variances, the datasets were subjected to a two-way Analysis of Variance (ANOVA). The statistical model was explicitly designed to test the main effects of the independent variables (‘Genotype’ and ‘Treatment’), as well as their interactive effect (‘Genotype × Treatment’). Following the ANOVA, multiple mean comparisons were performed using Tukey’s Honestly Significant Difference (HSD) post-hoc test, with statistical significance strictly defined at *p* < 0.05. All statistical analyses and graphical data visualizations were executed using GraphPad Prism 10.2.3.

The Stress Tolerance Index (STI) was calculated according to [33] following the summarization of the biological replicates, the Stress Tolerance Index (STI) for each evaluated trait was calculated using the mean values:

$$\:STI=\frac{{Y}_{p}\times\:{Y}_{s}}{{Y}_{p\_mean}^{2}}$$

In the formula, *Yp* and *Ys* represent the mean performance of a specific genotype under control and heat stress conditions, respectively. *Yp_mean* represents the grand mean of all evaluated genotypes under control conditions. Calculating STI from the genotype means, rather than individual sample values, ensures mathematical consistency with the population-level grand mean ($\:\stackrel{-}{Y}$p). The STI was deliberately chosen over simple relative normalization indices (e.g., Ys / Yp) because simple normalization mathematically biases tolerance rankings in favor of inherently small, low-yielding wild accessions (i.e., *S. pennellii*). By incorporating both control and stress performance (Yp x Ys), the STI formula effectively prevents this size-induced bias, strictly prioritizing genotypes that maintain both high absolute basal vigor and robust stress resilience.

Because MDA is negatively associated with stress tolerance, inverse values (1/MDA) were used to represent membrane stability prior to STI calculation. Heatmap visualizations and genotype clustering based on tolerance indices were generated using the *Seaborn* and *Matplotlib* libraries in Python (v3.9).

To evaluate the multidimensional heat stress response and generate an integrative, fuzzy logic-based ranking of the genotypes’ overall thermotolerance, Membership Function Values (MFV) were calculated based on the previously determined STI scores. The MFV (Uij) for each trait was computed using the following equation:

For positively associated traits:

$$\:{U}_{ij}=\frac{{X}_{ij}-{X}_{j\_min}}{{X}_{j\_max}-{X}_{j\_min}}$$

For negatively associated traits (i.e., MDA):

$$\:{U}_{ij}=1\div\frac{{X}_{ij}-{X}_{j\_min}}{{X}_{j\_max}-{X}_{j\_min}}$$

where U_ij_ is the membership function value of the j^th^ trait for the i^th^ genotype. X_ij_ represents the specific STI value of the j^th^ trait for the i^th^ genotype. X_*j_min*_ and X_*j_max*_ denote the minimum and maximum STI values observed for the j^th^ trait across all evaluated genotypes, respectively. Finally, the individual U_ij_ values across all evaluated physiological and biochemical traits were averaged to yield a single cumulative MFV for each genotype. A higher cumulative MFV indicates a superior, more balanced, and highly coordinated thermotolerance strategy.

Crucially, this equation functions as a Min-Max normalization technique, mathematically standardizing all trait indices into a strictly bounded, unitless range of 0.0 to 1.0. This transformation eliminates potential statistical bias caused by the inherently different absolute scales and variances of the evaluated physiological and biochemical parameters, ensuring that each trait contributes equitably to the final cumulative MFV score.

To visualize the functional relationships and rewiring among the morphological, physiological, and biochemical traits, a comparative correlation network analysis was conducted. The network graphs were constructed based on Pearson correlation coefficients (threshold |r| > 0.5) utilizing the *NetworkX* and *Matplotlib* libraries in Python (v3.9). The correlation network analysis was employed as an exploratory topological tool to visualize macroscopic, system-wide shifts in trait co-regulation, rather than to test isolated pairwise hypotheses. To ensure the robustness of the visualized network and prevent visual clutter from biologically negligible associations, a stringent effect-size threshold (Pearson’s |r| ≥ 0.5) was applied. While multiple testing corrections (e.g., False Discovery Rate) are standard for high-throughput datasets, they were deliberately omitted in this targeted network to prevent the inflation of Type II errors, which would otherwise mask genuine, biologically established linkages among inherently interconnected physiological and biochemical parameters.

The complete dataset containing all computed STI scores and individual membership function values (U_ij_) for each evaluated parameter across the thirteen genotypes is provided in Supplementary Table S1.

## Results

Two-way ANOVA revealed highly significant main effects for both ‘Treatment’ and ‘Genotype’, as well as a significant ‘Genotype × Treatment’ interaction, across all evaluated morphological, physiological, and biochemical parameters (Supplementary Table S2).

### Impact of heat stress on plant growth and water status

**Fig. 1:**
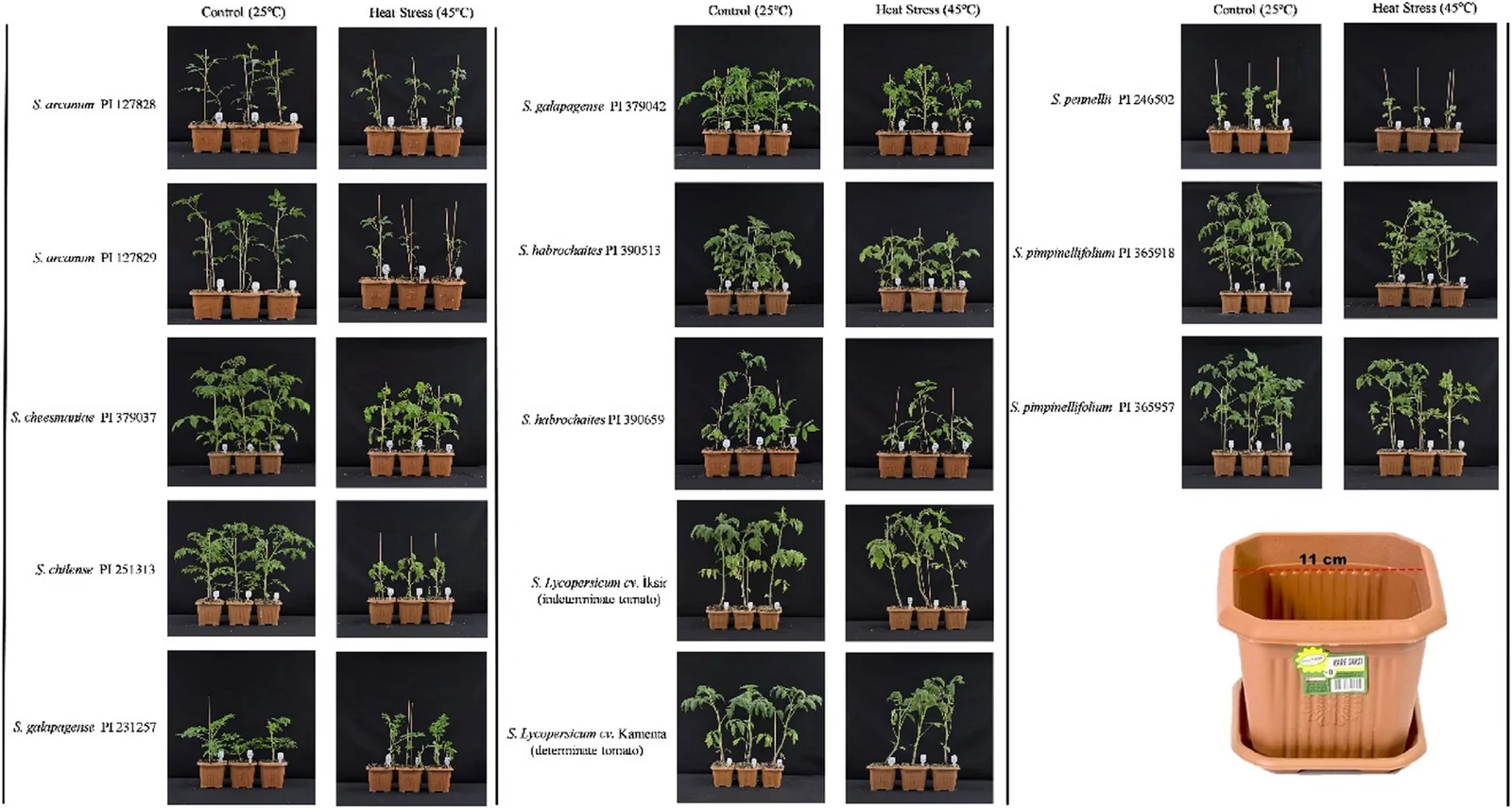
Phenotypic responses of 13 *Solanum* genotypes under control and heat stress conditions. Representative plants are shown under control (25/20°C, day/night) and heat stress (45 °C) within each genotype panel. The empty pot serves as a scale reference (rim diameter = 11 cm). Heat-sensitive genotypes exhibit wilting, chlorosis, and growth suppression, whereas tolerant accessions maintain vegetative integrity

Visual evaluation of the 13 tomato genotypes revealed clear phenotypic divergence under heat stress. Thermotolerant genotypes, particularly *S. lycopersicum* cv. İksir, maintained leaf turgor and structural integrity, whereas sensitive accessions such as *S. chilense* exhibited pronounced wilting, leaf rolling, and chlorosis (Fig. 1). Quantitative measurements confirmed these observations, demonstrating substantial variation in growth suppression among genotypes exposed to elevated temperatures.

**Fig. 2:**
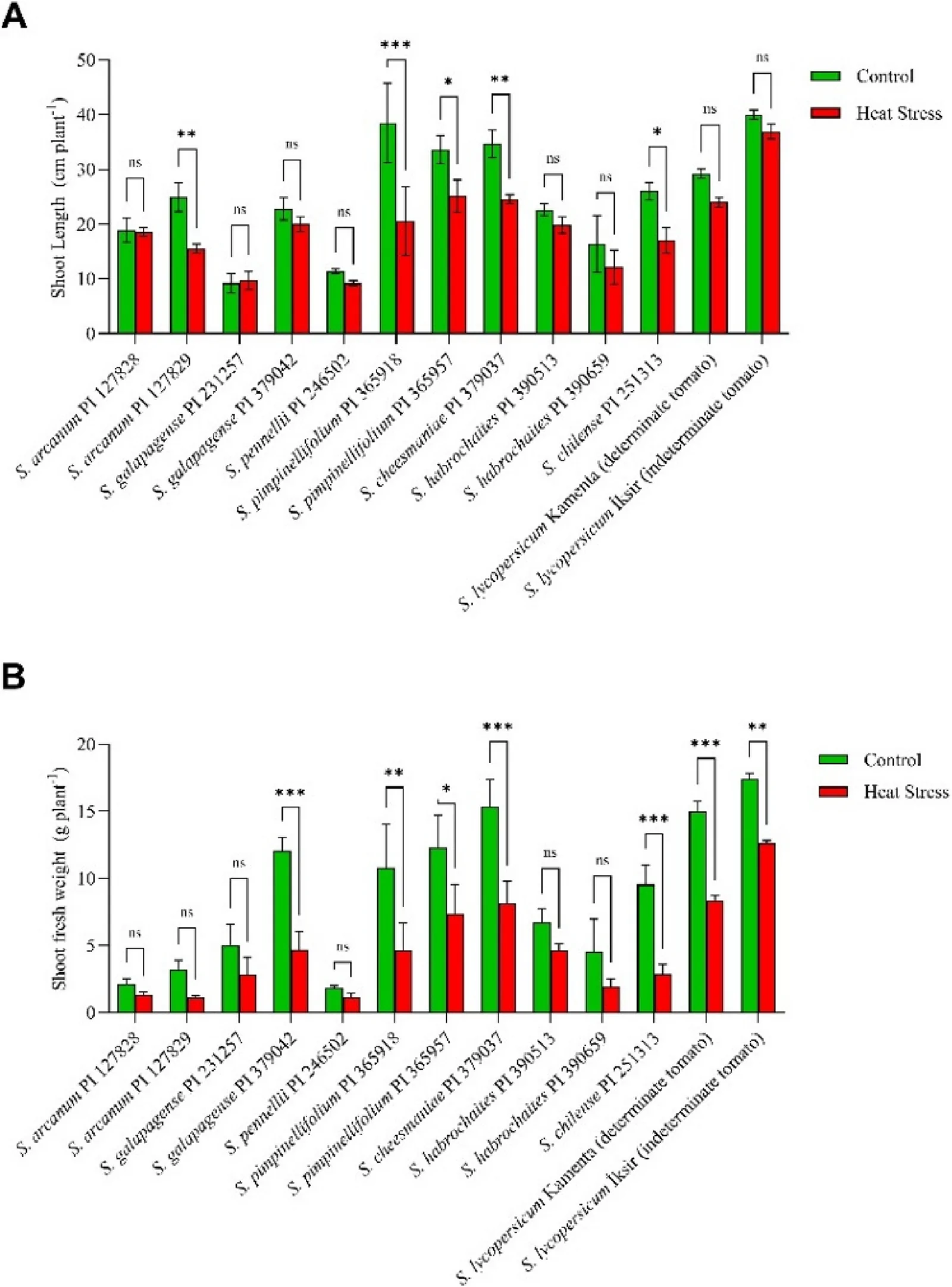
Vegetative growth responses under control and heat stress conditions. **A** Shoot length (cm) and (**B**) shoot fresh weight (g). Bars and whiskers represent the mean ± SE of each genotype (*n* = 5). Asterisks indicate significant differences between control and heat stress treatments based on Tukey’s HSD test (ns not significant, * *p* < 0.05, ** *p* < 0.01, *** *p* < 0.001)

Heat stress significantly affected vegetative growth parameters across all genotypes, although the extent of reduction varied markedly. Shoot fresh weight (SFW) emerged as the most responsive indicator of thermosensitivity. The commercial cultivar *S. lycopersicum* cv. İksir exhibited limited biomass reduction (27.6%), whereas *S. chilense* PI 251313 showed a severe decline (69.8%) relative to control conditions (Fig. 2B).

Shoot length followed a similar trend. Susceptible genotypes, including *S. arcanum* PI 127829 and *S. chilense* PI 251313, displayed pronounced growth inhibition, while *S. arcanum* PI 127828 maintained remarkable phenotypic stability, with less than 2% variation under stress. Collectively, *S. lycopersicum* cv. İksir and *S. arcanum* PI 127,828 demonstrated superior capacity to preserve plant height under hyperthermic conditions (Fig. 2A). Based on statistical analysis (Fig. 2), growth inhibition was most severe in *S. pimpinellifolium* PI 365918 and *S. cheesmaniae* PI 379037, which exhibited the most dramatic declines in shoot fresh weight. Conversely, *S. lycopersicum* cv. İksir maintained the highest absolute biomass, while accessions like *S. arcanum* PI 127828 demonstrated remarkable phenotypic stability relative to their controls.

**Fig. 3:**
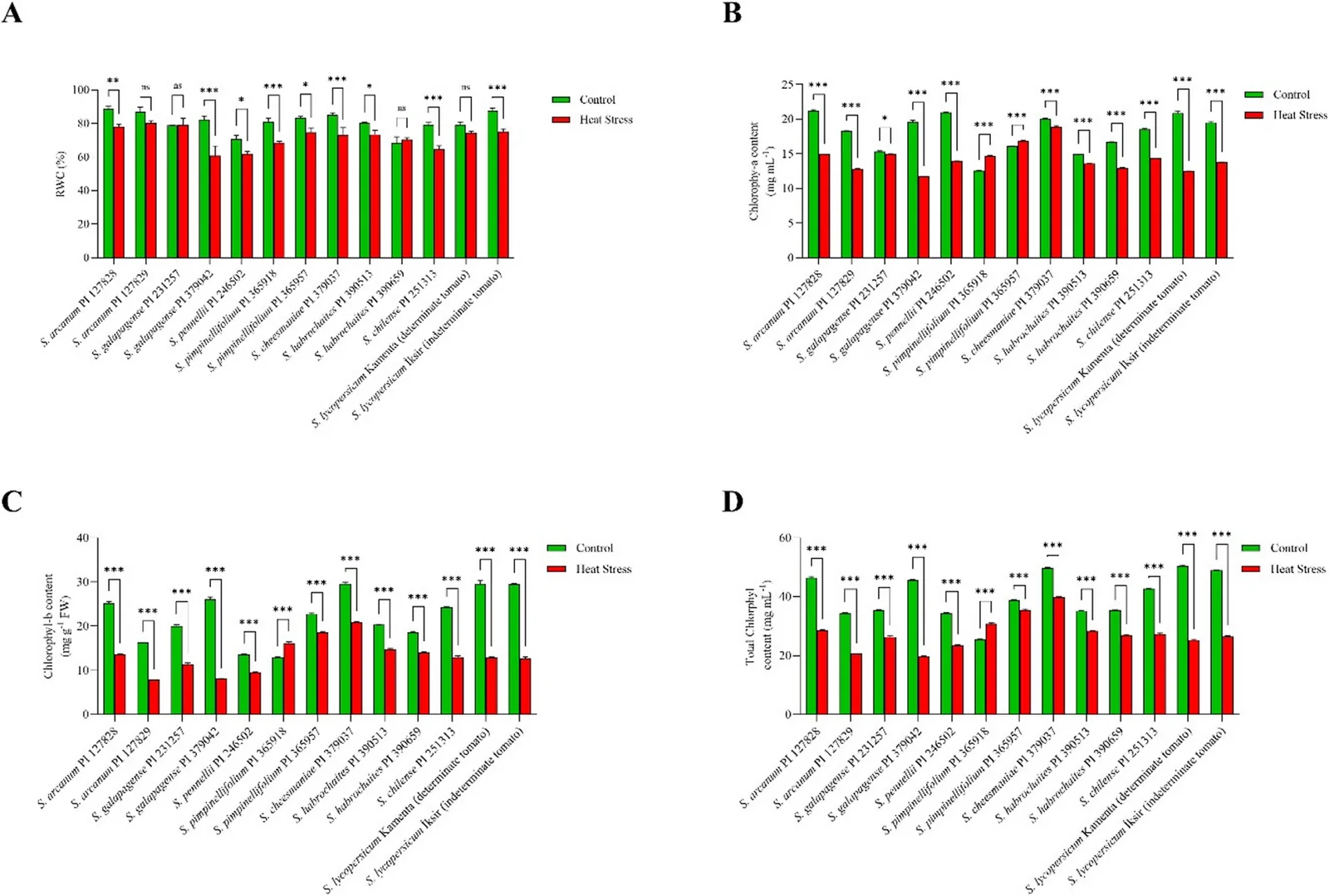
Effects of heat stress on leaf water status and photosynthetic pigment composition. **A** Relative Water Content (RWC), **B** chlorophyll a, **C **chlorophyll b, and (**D**) total chlorophyll. Bars and whiskers represent the mean ± SE of each genotype (*n* = 3). Asterisks indicate significant differences between control and heat stress treatments based on Tukey’s HSD test (ns not significant, * *p* < 0.05, ** *p* < 0.01, *** *p* < 0.001)

Heat stress significantly affected leaf hydration across the genotypes, revealing strong genotype-specific physiological adjustments. Specifically, RWC declined under heat stress conditions, although the magnitude of this reduction differed markedly among the evaluated genotypes. The greatest susceptibility was observed in *S. galapagense* PI 379042 and *S. chilense* PI 251313, which exhibited reductions of 26.1% and 18.4%, respectively. In contrast, *S. habrochaites* PI 390659 and *S. galapagense* PI 231257 maintained RWC values comparable to control conditions, indicating superior hydraulic stability. Interestingly, despite pronounced pigment degradation, the commercial cultivar Kamenta retained higher leaf water status (5.8% reduction) than the indeterminate cultivar İksir (14.1% reduction) (Fig. 3A).

### Heat-induced alterations in photosynthetic pigments

Total chlorophyll content declined in most genotypes, with the most pronounced reductions recorded in *S. galapagense* PI 379042 (− 56.6%) and *S. lycopersicum* cv. Kamenta (− 49.8%). This decrease was largely attributable to chlorophyll b degradation, reaching a maximum loss of 68.9% in *S. galapagense* PI 379042. Conversely, *S. pimpinellifolium* PI 365957 maintained pigment stability, showing only an 8.8% reduction in total chlorophyll. Notably, *S. pimpinellifolium* PI 365918 exhibited a compensatory increase in chlorophyll content (+ 20.8%) under heat stress (Fig. 3B-D).

### Membrane integrity and oxidative stress markers

Heat-induced membrane damage was assessed through malondialdehyde (MDA) accumulation, a reliable indicator of lipid peroxidation. A highly significant genotype-dependent response was observed, revealing marked divergence in oxidative stability among the genotypes.

Severe membrane damage was evident in specific wild accessions. *S. chilense* PI 251313 exhibited the greatest MDA increase (+ 108.5%), followed by *S. galapagense* PI 379042 (+ 64.9%) and *S. pennellii* PI 246502 (+ 25.3%) relative to control conditions.

**Fig. 4:**
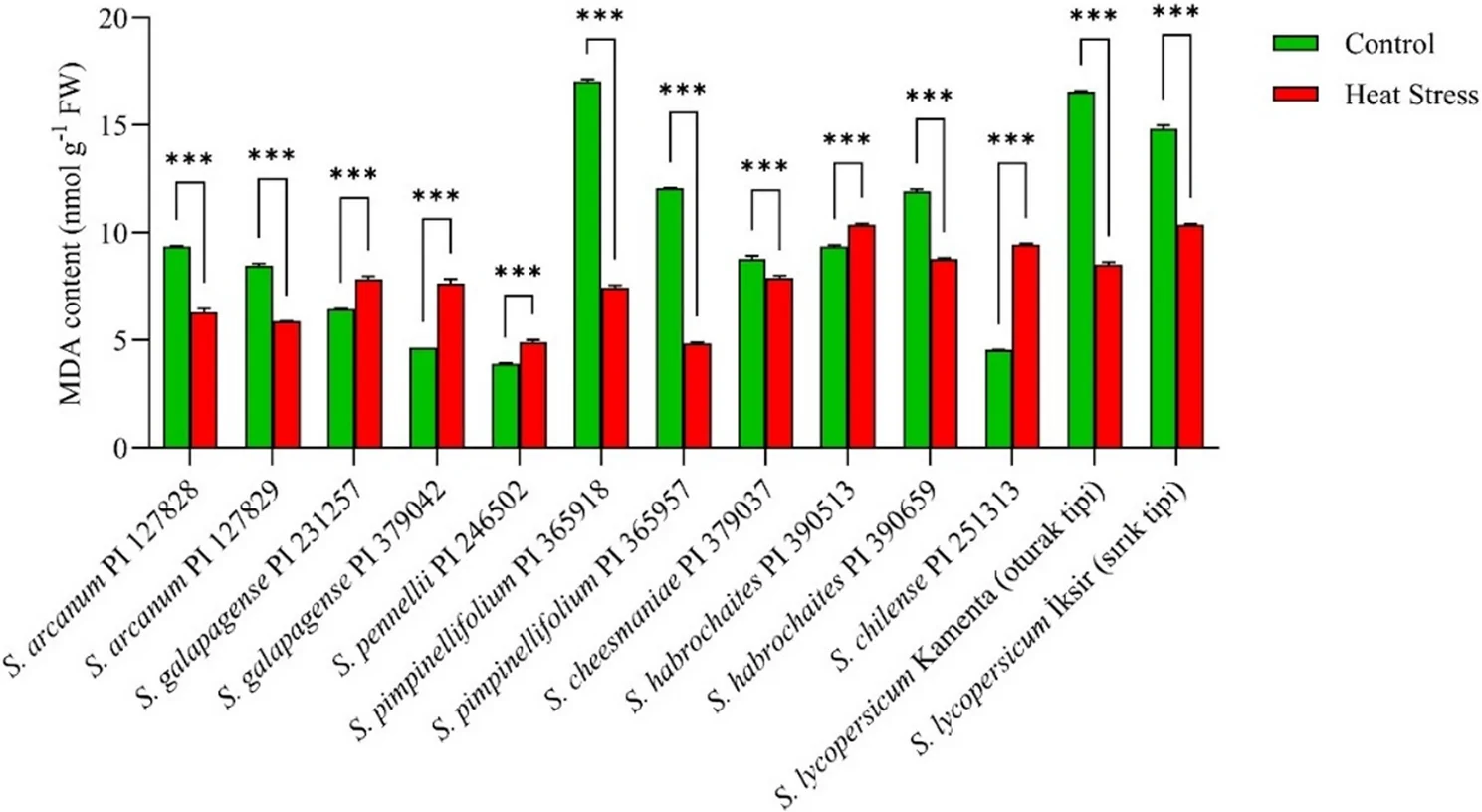
Membrane integrity under heat stress assessed by malondialdehyde (MDA) content. Bars and whiskers represent the mean ± SE of each genotype (*n* = 3). Asterisks indicate significant differences between control and heat stress treatments based on Tukey’s HSD test (ns not significant, * *p* < 0.05, ** *p* < 0.01, *** *p* < 0.001)

In contrast, several genotypes displayed stable or reduced MDA levels, reflecting enhanced membrane protection. *S. pimpinellifolium* PI 365957 showed the most pronounced reduction (− 59.9%), followed by *S. pimpinellifolium* PI 365918 (− 56.3%) and *S. lycopersicum* cv. Kamenta (− 48.4%). The indeterminate cultivar *S. lycopersicum* cv. İksir also maintained membrane stability (− 30.0%). The reduced MDA accumulation in these genotypes is consistent with their elevated antioxidant enzyme activities (Sect. Osmotic adjustment and antioxidant defense mechanisms), suggesting coordinated ROS detoxification and effective preservation of membrane integrity during hyperthermia (Fig. 4).

### Osmotic adjustment and antioxidant defense mechanisms

**Fig. 5:**
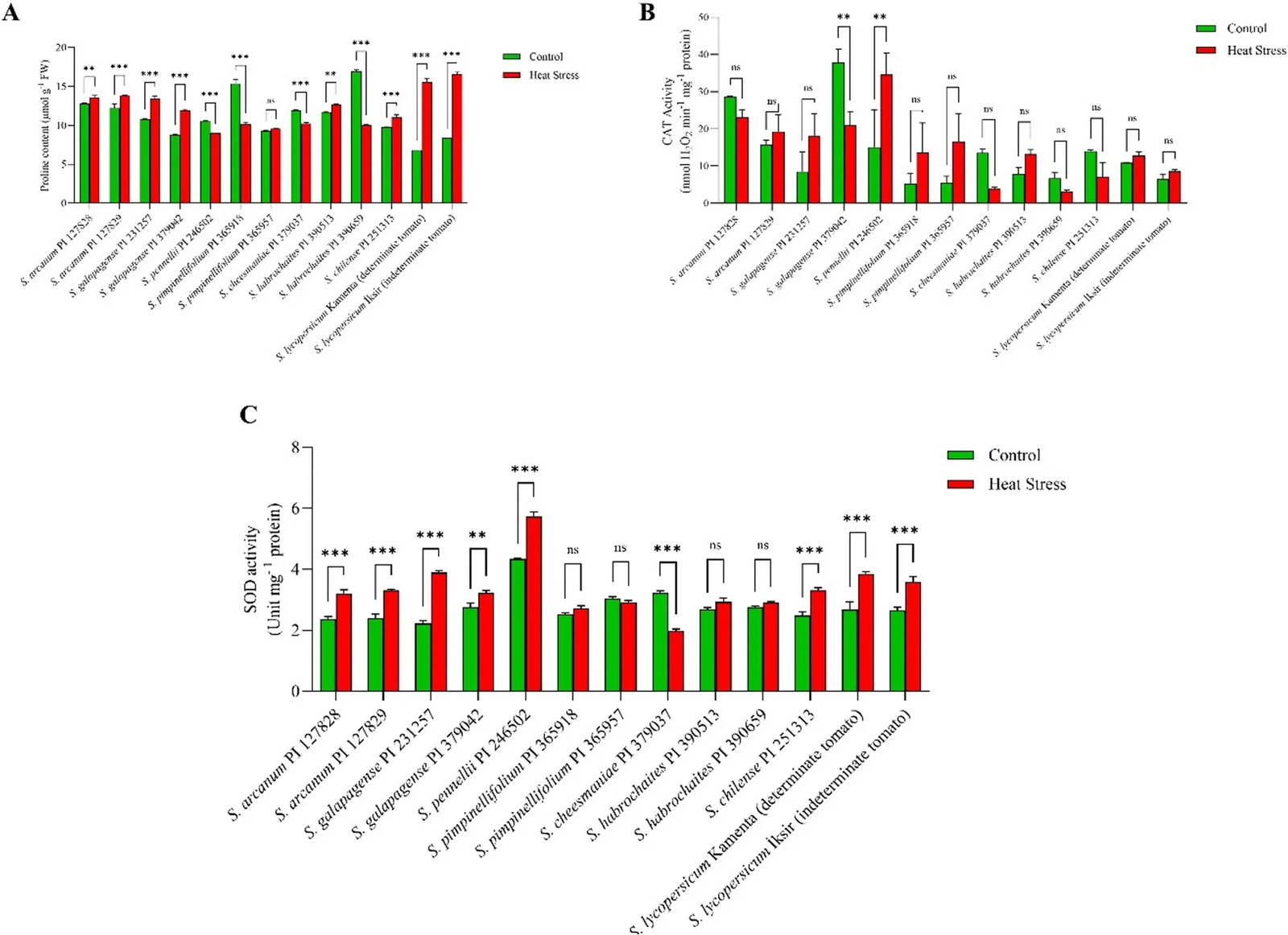
Osmotic and antioxidant defense responses under heat stress. **A** Proline content, **B** catalase (CAT) activity, and (**C**) superoxide dismutase (SOD) activity. Bars and whiskers represent the mean ± SE of each genotype (*n* = 3). Asterisks indicate significant differences between control and heat stress treatments based on Tukey’s HSD test (ns not significant, * *p* < 0.05, ** *p* < 0.01, *** *p* < 0.001)

Heat stress induced significant genotype-dependent modulation of compatible solutes and antioxidant enzymes. Proline accumulation, a key osmotic adjustment marker, was predominantly enhanced in cultivated genotypes. *S. lycopersicum* cv. Kamenta and cv. İksir exhibited substantial increases in proline content (+ 127.8% and + 98.2%, respectively). In contrast, several wild accessions, including *S. habrochaites* PI 390659 and *S. pimpinellifolium* PI 365918, showed significant reductions (− 40.7% and − 33.9%) (Fig. 5A).

Antioxidant enzyme responses displayed pronounced divergence. Catalase (CAT) activity was strongly induced in specific wild genotypes, particularly *S. pimpinellifolium* PI 365957 (+ 200.7%) and PI 365918 (+ 159.6%). Conversely, marked suppression was observed in *S. cheesmaniae* PI 379037 (− 70.4%) and *S. habrochaites* PI 390659 (− 54.4%) (Fig. 5B).

Superoxide dismutase (SOD) activity was generally elevated under heat stress, reflecting activation of primary ROS-scavenging pathways. The strongest induction occurred in *S. galapagense* PI 231257 (+ 75.4%) and *S. lycopersicum* cv. Kamenta (+ 44.1%). However, *S. cheesmaniae* PI 379037 exhibited a substantial decline (− 38.3%), further corroborating its susceptibility to oxidative damage (Fig. 5C).

### Multivariate analysis and genotypic ranking for heat tolerance

#### Stress Tolerance Index (STI)

**Fig. 6:**
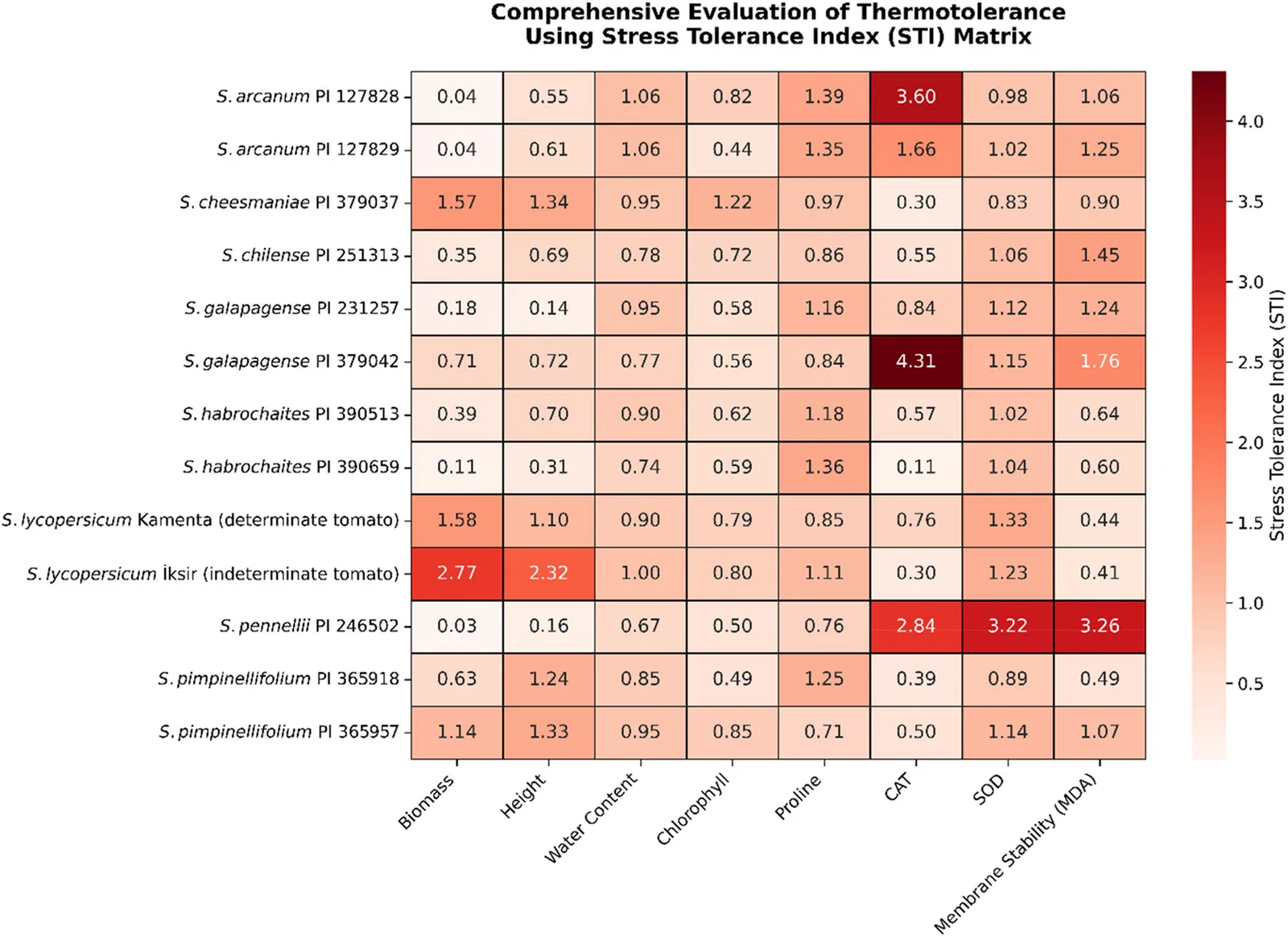
Stress Tolerance Index (STI)-based heatmap integrating growth, physiological, and biochemical traits. For MDA, inverse values (1/MDA) represent membrane stability. Darker shades indicate higher STI values

To integrate vegetative, physiological, and biochemical responses into a unified assessment of thermotolerance, a Stress Tolerance Index (STI) matrix was constructed (Fig. 6). This multivariate framework enabled identification of genotypes maintaining stable performance across multiple biological levels.

For biomass-related performance, the indeterminate cultivar *S. lycopersicum* cv. İksir exhibited the highest STI value for shoot fresh weight (2.77), indicating superior vegetative thermotolerance. It was followed by *S. lycopersicum* cv. Kamenta (1.58) and *S. cheesmaniae* PI 379037 (1.57), suggesting sustained productivity under elevated temperatures. In contrast, lower biomass STI values observed in *S. arcanum* PI 127828 and *S. pennellii* PI 246502 likely reflect inherent growth habit rather than intrinsic stress susceptibility.

Trait integration further revealed genotype-specific tolerance strategies. While *S. lycopersicum* cv. İksir maintained a balanced performance across most parameters, several wild accessions exhibited selective strengths. Notably, the membrane stability index (derived from inverted MDA values) highlighted the protective capacity of *S. pimpinellifolium* PI 365957, consistent with its elevated antioxidant enzyme activity.

Heatmap clustering partitioned the germplasm into three distinct categories: (i) High-yielding tolerant genotypes, characterized by sustained biomass and effective osmotic adjustment (e.g., *S. lycopersicum* cv. İksir); (ii) Physiologically resilient wild types, exhibiting strong enzymatic defense and membrane stability despite moderate biomass (e.g., *S. cheesmaniae* PI 379037); and (iii) Sensitive genotypes, displaying uniformly low STI values across growth and biochemical traits.

It is noteworthy that while specific accessions such as *S. pennellii* PI 246502 exhibited exceptionally high isolated STI values for antioxidant activity (Fig. 6), this intense biochemical response was accompanied by severe vegetative penalties. In contrast, the prominence of *S. pimpinellifolium* PI 365957 emerges not from a single extreme trait, but from a balanced, highly coordinated multidimensional response (combining robust ROS scavenging with moderate growth maintenance), which is mathematically captured by its superior cumulative MFV score (Fig. 8).

#### Correlation network analysis: reorganization of physiological interactions under heat stress

**Fig. 7:**
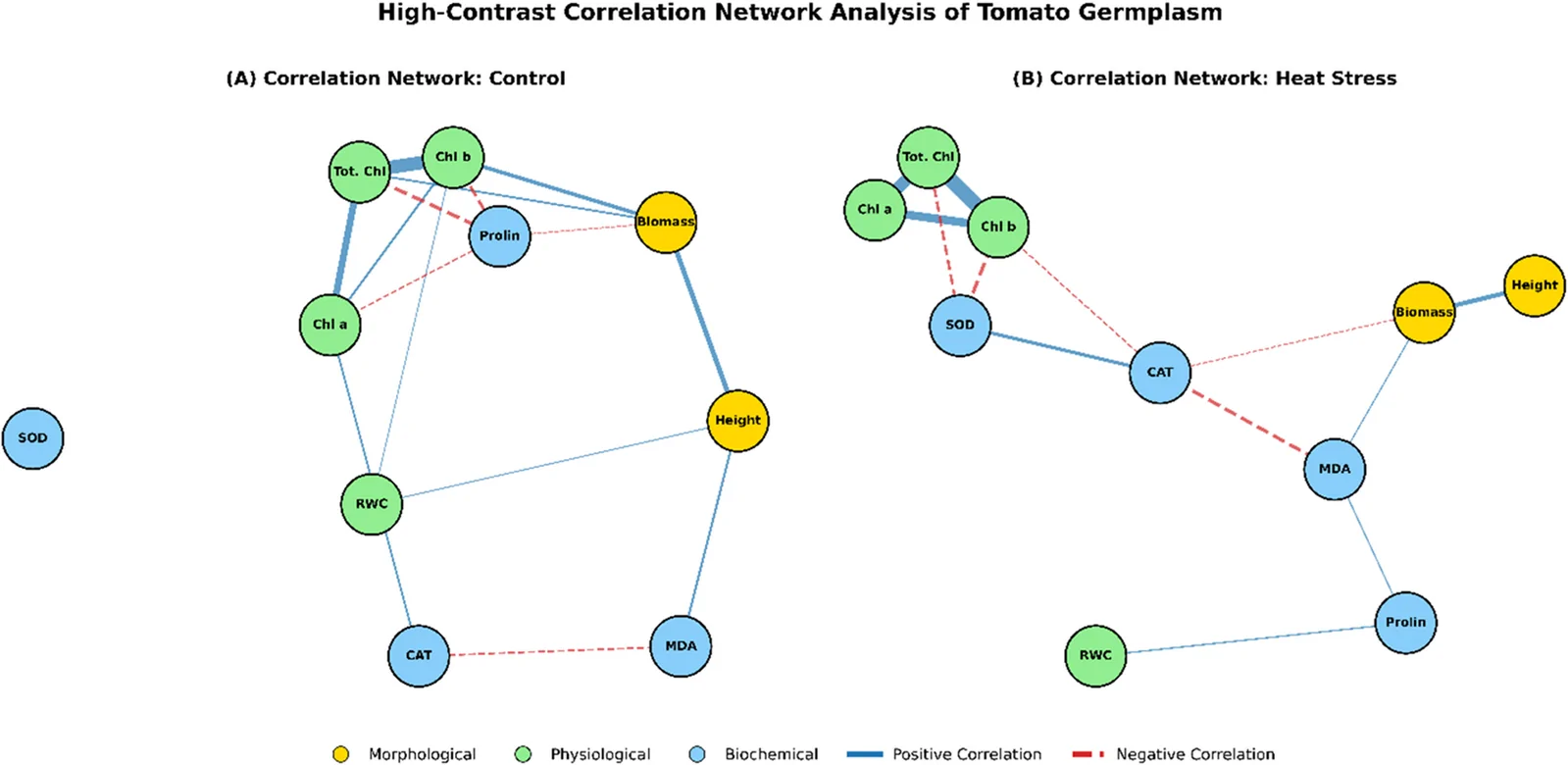
Correlation network analysis under (**A**) control and (**B**) heat stress conditions. Nodes represent measured traits and are color-coded by functional category. Edges denote significant Pearson correlations (|r| > 0.5), with thickness proportional to correlation strength

To examine functional shifts in trait interactions under heat stress, a comparative correlation network analysis was performed (Fig. 7). Network topology revealed a marked reconfiguration of physiological and biochemical associations between control and hyperthermic conditions.

Under control conditions (Fig. 7A), the network was dominated by strong positive correlations within the morphological cluster (biomass and height), which were closely linked to photosynthetic traits (total chlorophyll). This associative architecture indicates a growth-centered co-response pattern in which carbon assimilation and vegetative expansion are tightly coordinated. Antioxidant traits (CAT, SOD, proline) showed weak or peripheral connectivity.

In contrast, heat stress (Fig. 7B) induced a pronounced shift in network organization. A highly interconnected “defense module” emerged, characterized by strong positive associations among proline, CAT, and SOD. This coordinated clustering suggests synchronized osmotic adjustment and ROS detoxification in response to thermal stress. Simultaneously, significant negative correlations developed between MDA and growth-related traits (biomass, RWC), indicating that membrane lipid peroxidation directly constrains growth performance and water status. The attenuation of positive correlations between chlorophyll and biomass further suggests disruption of photosynthetic–growth coupling under stress.

#### Genotypic Ranking Based on Membership Function Value (MFV)

**Fig. 8:**
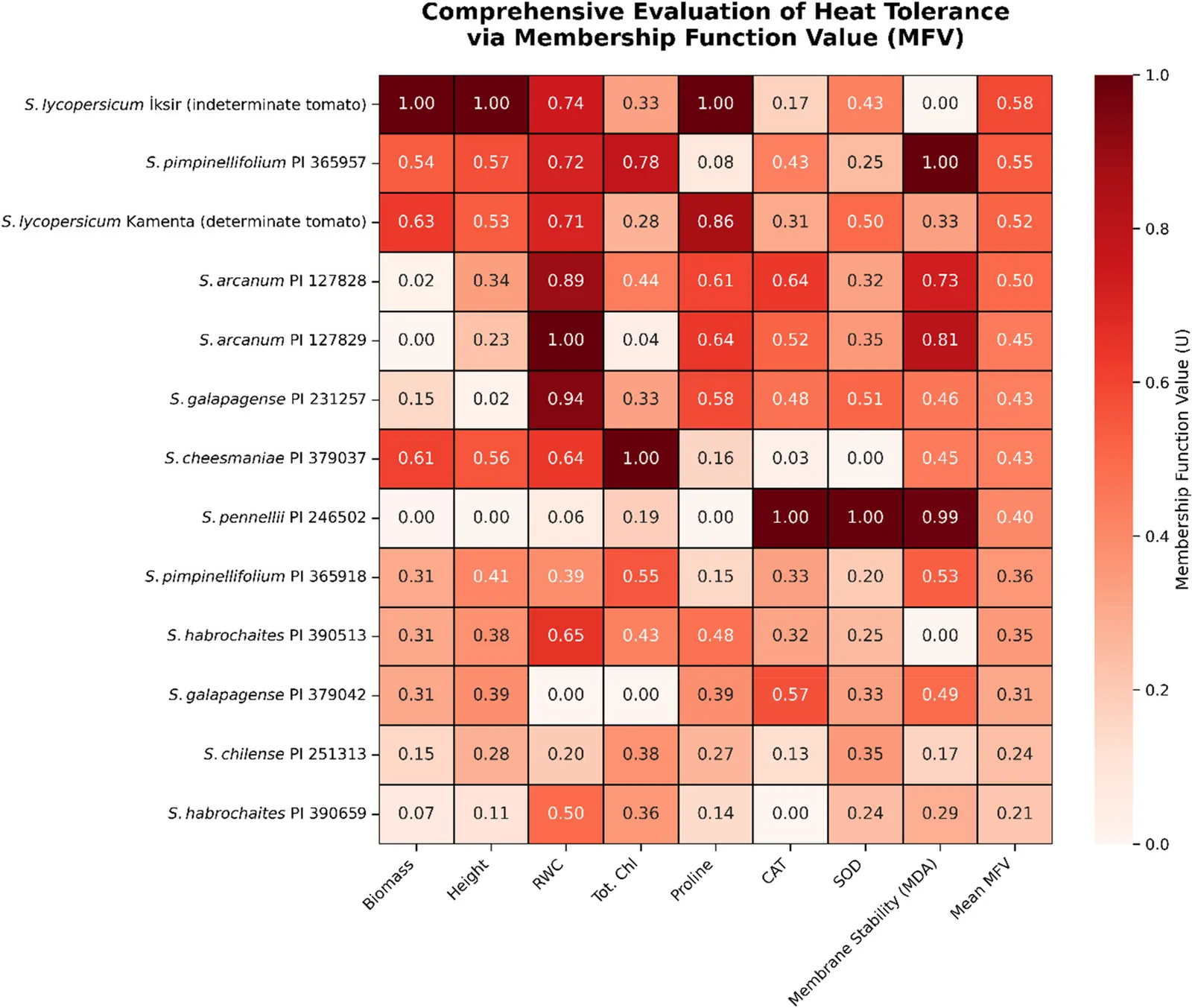
Membership Function Value (MFV)-based heatmap ranking under heat stress. The color scale represents normalized membership values (U), ranging from 0 (lowest performance) to 1 (highest performance). Genotypes are ordered in descending mean MFV, with the most thermotolerant positioned at the top. For MDA, inverse values (1/MDA) were used to represent membrane stability

To comprehensively evaluate the heat stress resilience of the germplasm and generate an integrative ranking of thermotolerance, the calculated STI and individual U_ij_ values (Supplementary Table S1) were utilized to construct cumulative, fuzzy logic-based MFV (Fig. 8). This multivariate approach successfully consolidates diverse physiological and biochemical parameters into a single composite score, where values approaching 1.0 indicate superior stress tolerance.

Crucially, because the MFV methodology integrates all evaluated morphological and biochemical traits with equal mathematical weight, this composite score explicitly rewards balanced, coordinated multi-trait performance rather than isolated phenotypic peaks. Based on mean MFV scores, the indeterminate cultivar *S. lycopersicum* cv. İksir ranked highest (Mean MFV = 0.584), reflecting stable biomass retention, coordinated antioxidant activation (SOD), and effective osmotic adjustment. It was followed by *S. galapagense* PI 231257 and *S. pimpinellifolium* PI 365957, which exhibited strong antioxidant performance and enhanced membrane stability, respectively.

In contrast, *S. cheesmaniae* PI 379037 and *S. chilense* PI 251313 recorded the lowest MFV values (< 0.241), indicating limited integrative resilience despite occasional strength in isolated traits. Their reduced composite scores corresponded with pronounced biomass loss and elevated membrane damage.

Overall, MFV clustering confirms that while commercial breeding has achieved substantial agronomic thermotolerance (as exemplified by *S. lycopersicum* cv. İksir), specific wild accessions harbor superior biochemical attributes that represent valuable targets for trait pyramiding and introgression in future heat-resilient breeding programs.

Based on the comprehensive MFV rankings and physiological strategies observed within this specific panel, the 13 evaluated genotypes can be explicitly allocated into three preliminary functional categories: (1) Growth-centric Tolerant (maintaining vegetative stability: *S. lycopersicum* cv. İksir, Kamenta, *S. pimpinellifolium* PI 365957, *S. cheesmaniae* PI 379037); (2) Defense-centric Tolerant (prioritizing biochemical survival:, *S. arcanum* PI 127828, *S. arcanum* PI 127829, *S. galapagense* PI 231257, *S. pennellii* PI 246502); and (3) Highly Susceptible (exhibiting systemic failure: *S. pimpinellifolium* PI 365918, *S. habrochaites* PI 390513, *S. galapagense* PI37904, S. chilense PI 251313, *S. habrochaites* PI 390659). However, we emphasize that this classification serves primarily as a descriptive framework specific to the tested germplasm, rather than a generalized biological model. Broader validation utilizing a substantially larger and more genetically diverse collection of tomato accessions is necessary to confirm the universal applicability of these functional categories across the *Solanum* genus.

A particularly intriguing finding of this study is the profound divergence observed between two accessions belonging to the same wild species, *S. pimpinellifolium* PI 365957 and PI 365918. Despite their close genetic lineage, these accessions exhibited starkly contrasting cumulative MFV scores. This striking discrepancy highlights that thermotolerance is not merely an interspecific (between species) trait, but rather exhibits substantial intraspecific (within species) plasticity. Such high accession-level variability highlights the importance of localized evolutionary adaptations and suggests that screening broad germplasm collections at the accession level, rather than relying on species-wide generalizations, is critical for identifying true climate-resilient donors.

## Discussion

The accelerating pace of global warming poses a major challenge to sustainable vegetable production, with heat stress emerging as one of the most critical abiotic constraints limiting tomato yield stability worldwide [34–36]. The comprehensive evaluation of thirteen *Solanum* genotypes in the present study demonstrates that thermotolerance is not a uniform trait within the genus; rather, it reflects pronounced phenotypic and physiological divergence shaped by distinct adaptive strategies.

A central finding of this work is the identification of a functional growth–defense duality underlying genotype-specific heat responses. The commercial cultivar *S. lycopersicum* cv. İksir maintained vegetative vigor and biomass stability under hyperthermic conditions, indicating breeding-driven optimization toward sustained vegetative performance. However, this stability was associated with comparatively moderate activation of specialized defense pathways. In contrast, the wild relative *S. pimpinellifolium* PI 365957 exhibited stronger antioxidant activation and enhanced membrane protection, reflecting a more defense-oriented strategy. These contrasting patterns suggest that domestication and selection for yield-related traits may have altered the regulation or plasticity of certain stress-responsive pathways that remain robust in wild germplasm [37–40].

Across all evaluated genotypes, exposure to extreme heat resulted in significant reductions in shoot height and fresh weight. Such growth inhibition is consistent with temperature-induced disruption of cell division, elongation, and carbon assimilation, frequently associated with altered microtubule dynamics [41–43]. Beyond these direct effects, the observed suppression likely reflects broader metabolic reprogramming. Reallocation of resources from primary growth toward protective functions aligns with the established framework of the growth–defense trade-off [44–47].

Under severe heat stress, plants redirect assimilates and energy from biomass accumulation toward survival pathways, including the synthesis of protective proteins, antioxidant enzymes, and compatible solutes [46, 48]. Within this context, *S. lycopersicum* cv. İksir retained a substantial proportion of its biomass, suggesting balanced resource allocation that permits both defense activation and continued growth. In contrast, the sensitive accession *S. chilense* exhibited pronounced growth inhibition and structural deterioration, indicating either insufficient defensive capacity or a metabolic cost exceeding its ability to sustain basal growth functions [49–52].

Physiological stability under heat stress was further differentiated by the ability of genotypes to maintain leaf relative water content (RWC) and photosynthetic pigment pools. Elevated temperatures disrupt chloroplast ultrastructure-particularly thylakoid membranes-and accelerate chlorophyll degradation, contributing to the chlorosis observed in sensitive accessions [53–58]. In contrast, tolerant genotypes such as *S. pimpinellifolium* PI 365957 preserved total chlorophyll content, indicating sustained integrity of the light-harvesting system and photosynthetic potential under thermal stress [59]. The maintenance of stable chlorophyll levels in tolerant accessions suggests enhanced photoprotective or adaptive pigment regulation mechanisms, a critical trait for sustaining photosynthetic efficiency under thermal stress [60, 61]. The preferential degradation of chlorophyll *b* over chlorophyll *a* observed in sensitive genotypes could be a critical photoprotective mechanism rather than a mere symptom of thermal damage [62]. Because chlorophyll *b* is primarily localized within the peripheral light-harvesting complexes (LHCII), its targeted degradation effectively reduces the antenna size of Photosystem II. This structural down-regulation minimizes the absorption of excess excitation energy when downstream carbon assimilation is heat-inhibited, thereby preventing severe photo-oxidative injury [63]. Conversely, the compensatory increase or strict maintenance of chlorophyll content in highly tolerant accessions, such as *S. pimpinellifolium* PI 365957, signifies a robust “stay-green” phenotype [64]. This phenomenon likely reflects a reactive structural remodeling of the thylakoid membranes, which not only sustains photoprotection but also preserves a high density of functional reaction centers, enabling the rapid resumption of photosynthetic activity once the acute heat stress is alleviated.

Similarly, higher RWC in these genotypes reflects effective osmotic adjustment [34, 65]. Accumulation of compatible solutes supports cellular water retention and turgor maintenance despite increased evaporative demand [66], thereby sustaining stomatal function and vegetative growth [67, 68]. The coordinated maintenance of pigment stability and hydration thus provides a mechanistic basis for the superior performance observed in resilient wild accessions and selected cultivars such as *S. lycopersicum* cv. İksir.

Mitigation of oxidative damage represents another critical determinant of thermotolerance. Coordinated activation of antioxidant enzymes and accumulation of compatible solutes constitute a central protective strategy under hyperthermic conditions [69]. Tolerant genotypes exhibited significant increases in superoxide dismutase (SOD) and catalase (CAT) activities, accompanied by enhanced proline accumulation and reduced MDA content. The observed elevations in MDA content among sensitive genotypes indicate intensified ROS-mediated lipid peroxidation and compromised membrane integrity under heat stress, a phenomenon widely recognized in susceptible plant models [61, 70]. Interestingly, while an accumulation of MDA is the classical hallmark of stress-induced membrane damage in sensitive crops, our data revealed an atypical, significant decrease in MDA content among specific highly tolerant wild accessions under severe heat stress. Although seemingly counterintuitive, we hypothesize that this reduction could be physiologically explained by extreme adaptive mechanisms unique to wild germplasm. While specific lipid profiling and RCS quantification were beyond the experimental scope of the present study, current literature suggests two potential pathways. First, under at gradually increasing thermal stress (45 °C), tolerant genotypes are known to rapidly remodel their membrane lipid composition, increasing the ratio of saturated to unsaturated fatty acids to maintain membrane thermostability [71, 72]. Because MDA is primarily a secondary product of *polyunsaturated* fatty acid peroxidation, this heat-induced membrane saturation inherently depletes the substrate required for MDA formation. Furthermore, recent evidence in extremophile plant models, such as halophytes, highlights the critical dynamics of reactive carbonyl species (RCS), including MDA, under severe abiotic stress [73]. In this context, the decreased MDA levels in our tolerant wild accessions likely reflect a hyperactive RCS detoxification capacity; rather than passively accumulating oxidative byproducts, these robust genotypes actively and rapidly scavenge reactive carbonyls to prevent cellular toxicity. However, because these specific biochemical pathways were not empirically quantified in our experimental design, this literature-based hypothesis remains speculative. Future targeted studies directly profiling membrane lipid saturation kinetics and RCS detoxification pathways are strictly required to validate these mechanistic assumptions in wild *Solanum* accessions. This suggests a highly dynamic, rather than purely degradative, lipid metabolism in wild *Solanum* relatives. Beyond its osmotic role, proline functions as a reactive oxygen species (ROS) scavenger, redox buffer, and stabilizer of proteins and cellular structures under stress [74–77]. In certain genotypes, diminished activities of specific antioxidant enzymes under severe stress suggest an impaired ROS detoxification capacity. Conversely, the accumulation of specific osmolytes without parallel enzymatic spikes indicates a reliance on alternative protective mechanisms [75, 78]. This suggests a synchronized, genotype-specific interplay between osmotic adjustment and ROS detoxification in response to thermal stress. Correlation network analysis further revealed a heat-induced reorganization toward a tightly connected “defense module,” characterized by strong positive associations among proline, CAT, and SOD. This restructuring indicates an integrated homeostatic adjustment prioritizing cellular protection overgrowth processes. In contrast, the sensitive accession *S. cheesmaniae* displayed reduced antioxidant activity under stress, suggesting impaired ROS detoxification and providing a mechanistic basis for its susceptibility [10, 79, 80].

Because plant responses to heat stress are inherently multidimensional, reliance on single parameters can lead to incomplete assessments of tolerance [81–83]. The use of integrative approaches such as the Stress Tolerance Index (STI) and Membership Function Value (MFV) allowed normalization of growth, water status, and biochemical defense traits into a unified tolerance score [84–88]. This multivariate evaluation clearly differentiated the tested germplasm into tolerant (e.g., *S. lycopersicum* cv. İksir and *S. pimpinellifolium* PI 365957), moderately tolerant, and sensitive (e.g., *S. chilense* PI 251313) categories. Overall, the correlation network analysis visually demonstrates a dramatic transition from a growth-oriented associative structure under optimal conditions to a defense-oriented co-response architecture under heat stress. In this stress-induced state, tightly correlated antioxidant and osmolyte responses become prominent features of thermotolerant phenotypes, reflecting their basal role in maintaining cellular homeostasis [89–91]. Such hierarchical classification, reflecting the integrated physiological profile rather than isolated traits, provides a practical framework for breeding strategies under climate change scenarios. While cv. İksir emerges as a promising vegetative-stage candidate for heat-prone regions, *S. pimpinellifolium* PI 365957 represents a valuable potential donor for the introgression of abiotic stress tolerance traits. However, any direct implementation in climate-resilient breeding programs must remain contingent upon rigorous future validation of these lines under variable field conditions and across reproductive developmental stages. While robust vegetative defense -such as that observed in *S. pimpinellifolium* PI 365957- constitutes a prerequisite for plant survival under abiotic stress, the degree to which such defense mechanisms translate into reproductive thermotolerance, or conversely impose penalties on yield-related traits, remains unresolved [92]. The pronounced genotype-dependent variation in key reproductive parameters under long-term mild heat observed in wild tomato species suggests that the functional linkage between early vegetative resilience and reproductive performance must be validated in mature plants through integrative physiological and genetic analyses [20, 93]. The findings of previous studies highlight that reproductive thermotolerance in tomato is governed by multiple temperature‑specific QTLs, and their observed strong genotype‑dependent reductions in fruit set and pollen viability under escalating heat regimes further underscore the need to integrate such temperature‑sensitive loci into breeding frameworks aimed at stabilizing reproductive performance under climate‑induced heat stress [94].

The feasibility of utilizing wild relatives in breeding programs is fundamentally dictated by their phylogenetic proximity to cultivated varieties [95]. In this regard, *S. pimpinellifolium* -the closest wild ancestor to *S. lycopersicum* within the red-fruited *Lycopersicon* clade- offers a distinct evolutionary advantage. Unlike more distant, green-fruited relatives such as *S. chilense* or *S. peruvianum*, introgressions from *S. pimpinellifolium* generally do not suffer from severe post-zygotic reproductive barriers or extreme linkage drag [96]. This high degree of phylogenetic and reproductive compatibility makes the targeted transfer of its multi-stress adaptive alleles (e.g., enhanced ROS scavenging and osmotic adjustment) highly feasible for modern breeding pipelines, ensuring that thermotolerance can be introgressed without compromising the agronomic integrity of elite cultivars.

Importantly, *S. pimpinellifolium* PI 365957 also carries the *Ph-3* gene conferring resistance to late blight caused by *Phytophthora infestans* [97], underscoring its multi-trait breeding value. The convergence of abiotic thermotolerance and established biotic resistance within a single wild accession highlights its strategic potential for developing climate-resilient tomato cultivars capable of withstanding both increasing temperatures and associated pathogen pressures. Beyond this unique combination of thermal and biotic resilience, such a comprehensive defensive repertoire is equally vital for tackling overlapping abiotic constraints. In the context of current climate change scenarios, extreme heat events rarely occur in isolation; they are frequently coupled with severe water deficits [98]. Therefore, an ideal candidate for climate-smart breeding must possess multi-stress tolerance. The prominent selection of *S. pimpinellifolium* PI 365957 in this study holds substantial promise for combined heat and drought environments. The superior defensive framework exhibited by this accession -specifically its massive proline-mediated osmotic adjustment and aggressive ROS scavenging through SOD and CAT- represents the fundamental physiological basis for drought tolerance as well (cross-tolerance) [99]. By tightly coordinating osmolyte and antioxidant networks, PI 365957 is biochemically pre-adapted to manage both thermal and osmotic cellular disruptions. While PI 365957 remains the most promising multi-stress donor among the evaluated germplasm, future research exposing these advanced introgression lines to concurrent heat and drought stress in field conditions will be imperative to fully validate its multi-stress agronomic viability.

## Limitations and future breeding perspectives

While the multivariate approach employed in this study provides critical insights into the growth-defense trade-offs under acute heat stress, certain limitations must be acknowledged. The evaluated parameters represent a specific subset of physiological and biochemical traits, predominantly focused on the vegetative stage. Key functional attributes, such as gas-exchange dynamics (stomatal conductance), photosystem II efficiency (Fv/Fm), and direct evaluations of reproductive competence (pollen viability, fruit set, and overall marketable yield), were not included. As true agronomic performance cannot be comprehensively assessed solely from vegetative indices, this lack of reproductive-stage evaluation remains a primary limitation of the current study and an essential target for future validation. Furthermore, the current study relies exclusively on morphological, physiological, and biochemical phenotyping. The absence of direct molecular validation, such as the targeted expression analysis of established heat stress marker genes via qRT-PCR or comprehensive transcriptomic profiling, represents a major limitation. Consequently, the distinct physiological and biochemical strategies identified herein provide a foundational blueprint, but strictly require future molecular validation to confirm the underlying genetic and regulatory networks. Finally, it is crucial to explicitly classify the nature of our applied thermal regime. The gradual escalation to 45 °C followed by a two-day exposure constitutes an acute heat stress (or heatwave) scenario. While resilience to such episodic, extreme thermal spikes is a critical component of climate adaptation, true field thermotolerance is a composite trait that also strictly requires adaptation to long-term, chronic mild heat stress. Because acute heat shock and chronic heat stress often trigger distinct physiological and molecular pathways, the adaptive strategies identified in this study specifically reflect acute survival mechanisms. Future longitudinal studies assessing these specific genotypes under prolonged, chronic thermal regimes are necessary to comprehensively determine their overall field resilience.Furthermore, while *S. pimpinellifolium* PI 365957 demonstrated superior biochemical defense, introgressing these adaptive traits into elite commercial backgrounds (such as cv. İksir) presents practical challenges due to potential linkage drag affecting yield. To overcome this, future breeding efforts should prioritize Marker-Assisted Backcrossing (MABC) and Quantitative Trait Loci (QTL) mapping. By utilizing molecular markers tightly linked to the specific antioxidant and osmotic regulatory networks identified here, breeders can achieve precision introgression, effectively uncoupling thermotolerance from undesirable wild traits and accelerating the development of climate-smart tomato varieties.

## Conclusion

Thermotolerance in tomato arises not from isolated physiological adjustments but from coordinated reorganization of growth, water balance, membrane integrity, and oxidative metabolism into an integrated stress-response architecture. Our integrative systems analysis demonstrates that acute heat stress drives a transition from growth-centered trait associations toward a synchronized stress-responsive configuration, establishing functional divergence among cultivated and wild genotypes as a network-level phenomenon.

The complementary strategies observed -biomass stability in *S. lycopersicum* cv. İksir and reinforced antioxidant coordination in *S. pimpinellifolium* PI 365957- underscore the importance of combining vegetative performance with robust cellular protection. Integrating these adaptive modules offers a rational pathway for developing tomato cultivars capable of sustaining productivity under increasingly frequent heat extremes.

By coupling multivariate indices with network-based modeling, this study provides a scalable framework for decoding complex stress-response architectures and guiding precision introgression of adaptive alleles into elite breeding backgrounds. Ultimately, these biochemical attributes represent valuable targets for trait pyramiding and introgression in future heat-resilient breeding programs. Based on our integrative MFV assessment under controlled 45 °C conditions, these results position *S. lycopersicum* cv. İksir as an immediate breeding candidate for vegetative heat resilience, while simultaneously identifying specific wild accessions (e.g., *S. pimpinellifolium*) as valuable donors for rootstock development. However, it is imperative to emphasize that these rankings apply exclusively to the acute vegetative stage. Subsequent validation under field conditions, reproductive phases, and combined multi-stress scenarios remains strictly essential to fully realize their broad breeding potential under future climate change realities.

## Supplementary Information

Supplementary Material 1.

Supplementary Material 2.

## Data Availability

The datasets used and/or analysed during the current study are available from the corresponding author on reasonable request.
